# The Progress of Medicine

**Published:** 1886-11

**Authors:** Hunter P. Cooper

**Affiliations:** Professor of Chemistry, Atlanta, Ga.


THE ATLANTA MEDICAL AND SURGICAL JOURNAL.

VOL. III. NOVEMBER, 1886. No. 9.

### Original Communications.

### THE PROGRESS OF MEDICINE.

AN ADDRESS DELIVERED AT THE OPENING OF THE ATLANTA MEDICAL COLLEGE, OCTOBER 7TH, 1886, BY HUNTER P. COOPER, M. D., PROFESSOR OF CHEMISTRY, ATLANTA, GA.

*Gentlemen:*

In casting about for a subject for this lecture, it occurred to me that a historical sketch of the progress of our profession might prove interesting. In studying the history of medicine, we are surprised to find how recent most of our knowledge is. Hippocrates, the father of medicine, did not know the difference between arteries and veins, and was totally ignorant of the functions of the nerves and their connection with the brain. From the creation of man to the 16th century medicine hung to the skirts of religion, sorcery and superstition. It had no distinct existence of its own. The priest healed the body by prayers and sacrifices to his deity, or the magician effected a cure by casting out devils. The ancient civilization of China and of Egypt, of Greece and of Rome, added but little to the advancement of the healing art.

Literature, painting, sculpture, architecture and engineering all flourished, under these different civilizations, to such an extent that to this day we still admire and seek in vain to surpass the achievements of antiquity. The Iliad still stands unrivalled as an epic poem. The beautiful creations of the chisel of Phidias have never been equalled. The exquisite beauty of Grecian architecture is copied at this day. The pyramids of Egypt still rank with the wonders of modern engineering. Ancient history teems with the names of famous statesmen, wise lawgivers, and profound philosophers. In medicine nothing was done; only two names have come down to us through the ages—Esculapius and Hippocrates. The skill of the former was limited to the dressing and healing of wounds, and prayer was his most efficient remedy. Hippocrates knew nothing of anatomy, physiology or therapeutics, but he laid down the principle which has in these latter days carried medicine to such a lofty eminence, namely, that "observation alone is the true basis of medicine." He taught his followers to abandon speculative theories and accurately observe the symptoms and course of disease. Had his teachings been followed, Grecian medicine might have ranked with Grecian philosophy. From the time of Hippocrates down to the 16th century, medicine was synonymous with quackery, priestcraft and sorcery. When the obscure mists of the middle ages began to clear away, and the human mind emerged from the dense darkness of ignorance and superstition, men began to see and think. The study of anatomy was the first direction in which progress took place. Mondini de Luzzi, professor of anatomy at Bologna, dissected the human body in 1315, the first time such a feat had been done in seventeen centuries. Little advance resulted from this, however, for nearly two centuries. In the 16th century the works of Hippocrates were translated and taught in the schools, and the promulgation of his methods began to bear fruit by putting medicine on a rational basis. The paramount importance of observation was recognized. As a consequence, the study of anatomy received a great impetus, and many important discoveries resulted. Vesalius, Fallopius, Ambrose Pare and Eustachius devoted themselves to dissection, and have rendered their names famous in medical history. Coincident with the growth and promulgation of anatomical knowledge, surgery began to take rank, though it was not until the middle of the eighteenth century that it was separated from its degrading association with the barber's art, and was practiced by educated men as a distinct profession.

During the 17th century anatomy and surgery made rapid strides—Harvey's discovery of the circulation of the blood being the most brilliant event of the age. The structure of the heart and lungs, of the muscular and nervous systems, and of the abdominal viscera, was carefully studied, and many false notions concerning the human body were overthrown. The progress of anatomy and surgery continued unabated in the 18th century and stimulated researches in physiology. During all this time medicine proper was still slumbering in the lap of empiricism. Physicians prescribed the most disgusting and unscientific remedies. Superstition was still deeply rooted in the minds of the people and the physicians. The advice and prescription of an old woman commanded as much respect as the counsel of the most learned doctor, and, to tell the truth, were just about as efficacious. I can give you no clearer idea of the state of medicine during the 18th century than by quoting a few extracts I made from a rare old book, published in London in 1749, entitled "A Complete English Dispensatory, by John Quincy, M. D." In making these quotations I run the risk of offending your finer sensibilities, but I will do so in order to show you how degraded medical practice was, and how little attention was bestowed on the action of remedies just a little more than 100 years ago.

"*Lumbrici Terrestres* (earth-worms).—These are often used in composition for cooling and cleansing the viscera. They are accounted much of the same nature with snails, but they seem to have more of an earthy or nitrous salt, which makes them afford parts more penetrating and detersive. They are good in inflammations and tubercles of the lungs, and are particularly used in such affections of the reins and urinary passages, where they cool and cleanse very much."

"*Limaces* (snails).—These seem to be much more in use now than formerly. They abound with a slimy, adhesive juice, and therefore are very good in weaknesses and consumptions, especially in children and tender constitutions. They are best boiled in milk or some such liquor. A syrup is the best preparation."

"*Syr. Limacum* (syrup of snails.—Take garden snails early in the morning while the dew is upon them, one pound. Take off their shells, slit them, and with half pound of fine sugar put into a bag, hang them in a cellar and the syrup will melt and drop through, which keep for use. It possesses, in the best manner, all the virtues of the snails."

"*Viperæ* (vipers).—The general opinion at present is that they are highly restorative and that they provoke venery." "The fat from the entrails" is thought to contain the active principle, for it is recommended "in dimness or decay of sight gently to rub the eyebrows with it."

"*Volatile Salt of Vipers.*—Many great and wonderful virtues are attributed to this salt, and it is reported to give relief, even in those diseases which are most refractory and difficult to cure, as apoplexies, lethargies, convulsions, palsies, and all other maladies believed to have their source from the brain." There is also a compound tincture of vipers.

"*Manus Hominis Mortui* (the hand of a dead man.)—This is supposed to be of great efficacy in dispersing scrofulous tumors. The part forsooth is to be rubbed with the dead hand for some time, and report furnishes us with many instances of cures done thereby. The sensation which stroking in that manner gives is somewhat surprising, and occasions a shuddering chilliness upon the part touched, which may in many cases put the fibres into such contractions as to loosen, shake off and dislodge the obstructing matter in which consists the cure."

Is it not incredible, gentlemen, that such absurd and ignorant practices were resorted to by educated (?) physicians only a hundred years ago? I am sure the therapeutics of the Hottentots would compare favorably with the practice of the middle of the 18th century.

Among other delectable resources the physician had in his warfare against disease, we find the following: "Wine of hog-lice" for jaundice, dropsy or any cachetic habit, and garden-snails boiled in the milk of a *red cow* for diseases of children.

Curiosity tempted me to look up the therapeutics of nux vomica, a drug highly and justly esteemed at the present day. The only notice that appears concerning it is the following: "Its principal use is to do mischief with by killing dogs and cats."

John Quincy, M. D., is dead and gone these many years. God rest his bones. He died perhaps at a ripe old age, with the sweet consciousness of having, by means of earth-worms, snails, snakes and hog-lice, snatched countless fellow-beings from the jaws of death.

Happily the latter part of the 18th century is adorned by many famous names. John Hunter, the great English surgeon; Percival Pott, also eminent in surgery, and last and greatest, the immortal Edward Jenner, who in 1798 demonstrated a method of preventing small-pox, that loathsome and fatal scourge, which, from the time of the Crusades to the discovery of vaccination, was the terror of the whole world.

The causes which led to the brilliant advances our profession has made in the 19th century may be summarized thus: 1st. The numerous discoveries in chemistry and physics. 2nd. The study of anatomy, physiology and pathology. 3d. The use of the microscope. 4th. Scientific experimentation. Medicine, for the first time in the world's history, was placed on a scientific basis. False notions concerning the structure of the human body and the functions of its organs faded away under the bright light of philosophical investigation. The anatomist's scalpel and the histologist's microscope gave us accurate information concerning all parts of the body. Improved methods of chemical analysis gave the physiologist the power to determine the composition of the fluids of the body, and by means of experiments on living animals the functions of the various organs were ascertained. The actions of drugs, both in health and disease, were studied in the same scientific way, and traditions, centuries old, regarding the treatment of disease, were discarded for more rational therapeutics.

Diseases were studied and classified, and the invention of instruments of precision aided in their diagnosis. Materia medica was enriched by the addition to its list of remedies of many valuable drugs, both from nature and from the chemist's laboratory. With increased knowledge of anatomy and improved methods of operating surgery made rapid strides. Let me recount some of the most important discoveries of this century.

*Vaccination.*—This discovery, made just at the close of the 18th century, did not bear any fruit until the 19th. Prior to the inoculation of vaccine virus, it is estimated that fully one-tenth of all deaths in Europe were due to the ravages of small-pox. When an epidemic arose in a city there was no protection, save flight, against the dread disease, and it raged unopposed until its appetite for human life was satiated by thousands of victims. It conquered proud armies and laid waste fair cities. It respected neither wealth, rank nor beauty, but with equal zest licked with its tongue of poison cheek of prince and cheek of beggar, jewelled queen and wrinkled hag.

A plain country doctor arose to meet this haughty destroyer, and the world hissed and sneered at his presumption, even as the Philistines of old jeered and laughed when the simple David appeared against their mighty warrior. In the monotonous routine of country practice, Edward Jenner observed that farmers and dairymen, who had contracted a sore on the hand from the cow's teat, were protected in some mysterious way from small-pox. Others had observed this before Jenner, but it was reserved for his master-mind to grasp the tremendous import of this simple fact, and by a process of reasoning and experiment convert it into the grandest benefit that has ever been conferred on suffering humanity.

*Anæsthesia.*—A little more than forty years ago, a quiet, unassuming physician, Crawford Long by name, lived in Jackson county, Georgia, and pursued there the practice of his profession. He noticed that a person who inhaled sulphuric ether for its intoxicating effects suffered no pain when in his drunken exhilaration he struck a blow with his bare fist upon a hard wall. He reasoned from this that surgical operations might be painlessly performed if the patient was brought profoundly under the influence of the drug. Acting on this idea, he etherized a patient and operated successfully. Unfortunately he has never obtained the recognition of the world at large as the discoverer of anæsthesia, because he did not at once publish his discovery, and the laurels were snatched from his brow by Drs. Jackson and Morton, who operated in 1846 on a patient under the influence of ether. Dr. Marion Sims, however, has proved, beyond doubt, that the credit of priority belongs to Dr. Crawford Long. Soon afterwards Dr. Simpson, of Edinburgh, discovered the anæsthetic property of chloroform. It is impossible to estimate the benefit which Dr. Long and Dr. Simpson have conferred on the human family. Not only has anæsthesia banished all the suffering connected with surgical operations, but it has rendered practicable and simple many operations which could not be undertaken prior to its discovery. By diminishing shock and by allowing the surgeon to proceed more slowly and carefully with his work, anæsthesia has greatly lessened the mortality of surgical operations.

*Ovariotomy.*—The propriety of removing ovarian cysts was discussed a century and a half ago, but it was reserved for Dr. Ephraim McDowell, of Kentucky, to first perform this operation; this he did in 1809 and saved his patient. His brilliant discovery has been the means of relieving the suffering and saving the lives of hundreds of women. For fifty years after McDowell's discovery, the operation languished, because the conditions on which success depended were but little understood, and the methods of performing it were imperfect. About twenty years ago, however, the operation began to grow in favor, and now it is performed almost daily in large city hospitals, and the mortality reduced to between 2 and 8 per cent.

In *chemistry and materia medica* wonderful advances have been made. To the chemist's art we owe our present knowledge of the properties of drugs and the isolation of their active principles. Quinine, morphine, atropine, strychnine, chloral, chloroform, ether, antipyrine, cocaine and a host of other most valuable remedies have been discovered during this century.

The introduction of *hypodermic medication* thirty years ago marks another era in medical progress. At first it was limited in its application, morphine alone being used. Now we give many other remedies by hypodermic injection. Its advantages are: 1st. Rapidity of effect. 2d. Certainty of effect. 3d. It causes no disturbance of the stomach. 4th. By hypodermic injection drugs can be administered to a patient who is unable to swallow. The hypodermic injection of morphine is the most certain, rapid and powerful means we have for the relief of pain. Nothing, however, is more dangerous than the indiscriminate use of the hypodermic syringe, for patients contract the morphine habit much more easily where the drug has been employed hypodermically than when administered by the mouth. The use of other drugs by hypodermic injection is also invaluable. Those of us who have had occasion to treat profound shock, or heart failure from any cause, or alarming hemorrhage, will remember with pleasure the priceless comfort our hypodermic syringe afforded us.

*Antisepsis.*—The germ theory of disease is an outgrowth of the perfection of the microscope. The microscope has revealed the presence of minute living organisms in the air we breathe, the water we drink, the food we eat, and in all fermenting or putrefying substances. We are surrounded and covered by myriads of these little microbes. A careful study of them by the microscope has enabled us to classify many of these microbes and to ascertain their functions. Different kinds of micro-organisms require different conditions for their growth and propagation—thus some thrive only in a saccharine or starchy soil, while others prefer an albuminous substance. By their growth and development micro-organisms give rise to fermentation and putrefaction, changing by their vital actions the chemical nature of the soil in which they grow. According to the germ theory of disease, all contagious and infectious diseases are due to the growth in the human body of some special micro-organism. Each disease is believed to have a special and distinct kind of microbe as its cause. The truth of this belief has been demonstrated for certain diseases, viz., charbon, Asiatic cholera, tuberculosis and hydrophobia, and it is highly probable that in time the microbian origin of all other contagious diseases will be demonstrated. This theory of disease was accepted by Mr. Lister in order to explain wound infection. He assumed the presence of poison-generating microbes in the air, water, bedding, on the surface of the human body; these microbes easily gain access to a wound, and by their presence set up either local inflammation or general blood-poisoning. In accordance with this view, Mr. Lister devised his antiseptic method of treating wounds. By the use of various carbolic solutions, he destroys all the germs which are already on the surgeon's hands and instruments, or which have gained access to the wound. He then dresses the wound in such a way that no germs can find their way to it without passing through a carbolic atmosphere which will prove fatal to them. Though Lister's method has been variously modified, his principle is recognized and carried out by nearly all the leading surgeons of the world.

Under antiseptic methods surgery has undergone a complete revolution. Operations, formerly considered almost inevitably fatal, are now performed daily, the parts often healing by first intention. Pyæmia, septicæmia, erysipelas and hospital gangrene are now almost unknown.

In obstetrics the introduction of antisepsis has lessened very materially the mortality of parturition; for instance, the mortality in the lying-in wards of the Vienna General Hospital was 28 per thousand twenty-five years ago; now it varies from 2 to 5 per thousand. These figures are from Prof. Carl Braun's statistics.

*Cocaine.*—Only two years ago the world witnessed another great discovery in medicine: I allude to the anæsthetic property of cocaine. In the latter part of 1884, Dr. Koller, of Vienna, discovered the remarkable anæsthetic effect of a solution of cocaine on the conjunctiva. Soon afterwards it was ascertained that it was not only anæsthetic to all mucous surfaces, but also to the skin and other structures when given by hypodermic injection. It is truly a most wonderful drug. Minor surgery is by its use greatly simplified. A few months ago Dr. Corning, of New York, devised a method by which major operations could be performed painlessly by cocaine. Briefly stated, his method consists in confining the blood by means of an elastic bandage to the part to be operated upon. Acting on this plan, Dr. Varick, of Jersey City, performed an amputation of the thigh on February 13th, 1886, using cocaine alone as an anæsthetic. The patient was perfectly conscious, and experienced no pain whatever until the bone was divided.

Time fails me, gentlemen, to speak in detail of the discoveries in pathology, physiology and therapeutics.

In its rapid growth medicine has thrown out numerous branches—ophthalmology, laryngology, gynecology, neurology, etc. Special study in these various branches has greatly increased our knowledge.

In conclusion, gentlemen, I will say that a review of medical history shows us that in no other profession is there so little room for genius. In medicine, plodding, untiring perseverance will inevitably win, and the man who trusts to genius will lag behind in the race for professional success. A country doctor discovered the preventive for small-pox. A country doctor performed the first ovariotomy. A country doctor discovered the anæsthetic property of ether. The country doctor, gentlemen, has wielded a powerful influence on the progress of medicine.

Cultivate habits of industry and habits of observation, and you will certainly achieve success, not only in your college career, but also in that broader arena, the world.

---

DOUBLE EMBRYO IN A SINGLE BLASTODERM.—Professor Legge, in a communication made to the Eustachian Society of Camerire, states that he has had the good fortune of meeting with, in a fowl's egg, at about the third day of incubation, two embryos in a single blastoderm, joined together at the summit. —*Medical News*.

